# The Blood–Brain Barrier, Oxidative Stress, and Insulin Resistance

**DOI:** 10.3390/antiox10111695

**Published:** 2021-10-27

**Authors:** William A. Banks, Elizabeth M. Rhea

**Affiliations:** 1 Department of Medicine, Division of Gerontology and Geriatric Medicine, University of Washington, Seattle, WA 98195, USA; wabanks1@uw.edu; 2 Geriatric Research Education and Clinical Center, Veterans Affairs Puget Sound Health Care System, Seattle, WA 98108, USA

**Keywords:** blood–brain barrier, oxidative stress, insulin resistance, diabetes mellitus, Alzheimer’s disease

## Abstract

The blood–brain barrier (BBB) is a network of specialized endothelial cells that regulates substrate entry into the central nervous system (CNS). Acting as the interface between the periphery and the CNS, the BBB must be equipped to defend against oxidative stress and other free radicals generated in the periphery to protect the CNS. There are unique features of brain endothelial cells that increase the susceptibility of these cells to oxidative stress. Insulin signaling can be impacted by varying levels of oxidative stress, with low levels of oxidative stress being necessary for signaling and higher levels being detrimental. Insulin must cross the BBB in order to access the CNS, levels of which are important in peripheral metabolism as well as cognition. Any alterations in BBB transport due to oxidative stress at the BBB could have downstream disease implications. In this review, we cover the interactions of oxidative stress at the BBB, how insulin signaling is related to oxidative stress, and the impact of the BBB in two diseases greatly affected by oxidative stress and insulin resistance: diabetes mellitus and Alzheimer’s disease.

## 1. Introduction

The blood–brain barrier (BBB) helps to separate the periphery from the central nervous system (CNS). This specialized network of brain endothelial cells (BECs) interacts with astrocytic endfeet, pericytes, neurons, and the basement membrane to form the neurovascular unit (NVU). BECs are connected by tight junctions (TJ) and express specific transporters to regulate substrate entry into and out of the brain. The brain relies on this network of cells to manage interactions with the circulation. BECs are constantly exposed to potential free radicals or stress-inducing elements such as cytokines and inflammatory cells through interactions with circulatory factors. BECs contain extra defense mechanisms against oxidative stress, including increased glutathione and related enzymes [1]. Endogenous (glutathione, lipoic acid, superoxide dismutase) and exogenous (vitamins, carotenoids, polyphenols) antioxidants exist for humans and there are differences in antioxidant defense systems across species. For example, humans cannot synthesize ascorbate and must acquire it from the diet, while most other mammals are able to produce ascorbate endogenously, with production levels corresponding to levels of oxidative stress [2]. 

Low amounts of reactive oxygen species (ROS) and reactive nitrogen species (RNS) are important regulatory mediators in many signaling processes. Oxidative stress occurs when there is an imbalance between the generation of ROS, RNS, metal ion homeostasis, and antioxidant defenses. Lipid peroxidation is a type of oxidative stress that occurs when cellular membranes, lipoproteins, and other lipids are exposed to oxidative substrates. DNA and protein oxidative damage due to oxidative substrates are other types of oxidative stress. When the level of oxidative stress is too high and/or antioxidant defenses are impaired or altered, oxidative damage occurs, and can be a primary culprit in diseases. Insulin resistance and apolipoprotein E (apoE) are also linked with oxidative stress and can impact the BBB. We will discuss the link between the BBB, oxidative stress, insulin receptor signaling, and two diseases closely associated with insulin resistance: diabetes mellitus (DM) and Alzheimer’s disease (AD).

## 2. Oxidative Stress at the BBB

**Figure 1:**
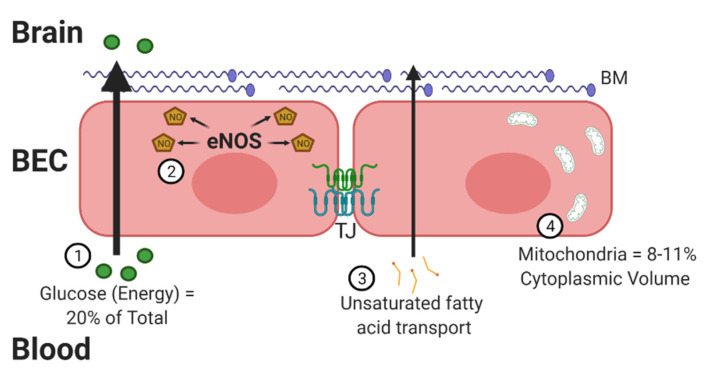
The blood–brain barrier (BBB) and oxidative stress. There are 4 distinct ways brain endothelial cells (BECs) are at a greater exposure to oxidative factors. First, these cells must transport high levels of glucose into the brain for energy. Glucose must be metabolized to energy-utilizing substrates within mitochondria, generating free oxygen radicals. Second, BECs generate high levels of nitric oxide (NO) through endothelial nitric oxide synthase (eNOS), required for intracellular signaling and regulation of vascular tone. Third, BECs must transport dietary lipids and fatty acids into the brain as an alternative source of energy and signaling. This creates an increased opportunity for the generation of lipid peroxidation. Lastly, BECs contain a greater number of mitochondria compared to peripheral endothelial cells. Mitochondria are the primary source of reactive species, including superoxide. In all, BECs require a critical counter-regulatory process to combat the oxidative factors in order to maintain a functioning BBB with preserved tight junction (TJ) proteins and basement membrane (BM). Figure generated using BioRender.

The BBB contains properties that can not only combat oxidative stress but also lead to the development of free radicals [3]. There are four critical traits that increase the susceptibility of the BBB to oxidative stress (Figure 1). The brain is a highly metabolic organ, utilizing approximately 20% of the body’s energy through oxygen consumption. A potential side effect of this high oxygen consumption is an increased risk for generating ROS products. The energy substrates must cross the BBB to reach the brain, prior to being metabolized locally. Second, the BBB utilizes nitric oxide (NO) to regulate vasodilation, which increases the opportunity for RNS. A fast response of the vasculature is especially important for the brain, which does not store glucose and relies on blood flow for nutrients. Dysregulation of NO synthase (NOS) enzymes, in particular endothelial NOS (eNOS), which occurs under high-glucose conditions, can lead to oxidative stress [4]. Next, neuronal membranes are largely made up of polyunsaturated fatty acids, such as docosahexaenoic acid (DHA). DHA must be transported across the BBB [5]. These fatty acids are susceptible to lipid peroxidation. BECs are particularly sensitive to lipid peroxides [6]. 

Compared to peripheral endothelial cells, BECs have a high concentration of mitochondria [7] to meet their energy needs, providing an opportunity for increased oxidative stress [8]. BECs were found to have mitochondrial contents of 8–11% of the cytoplasmic volume compared to 2–5% in non-BBB cells. Mitochondria serve as one of the primary sources for free radical generation due to the high percentage of oxygen consumed. The increased number of mitochondria also help in BBB maintenance. BEC mitochondria decrease in number with aging [9,10], but this does not necessarily decrease the amount of oxidative stress generated. In fact, oxidative stress is implicated in aging [11] and AD, as will be discussed at the end of this review. In addition to the mitochondrial respiratory chain contributing to the production of ROS, brain endothelium also contains NADPH oxidase (Nox) and xanthine oxidase, which contribute to ROS generation [6]. These four processes highlighted above make it critical for the BBB to have endogenous defense systems in place.

BECs can combat production of ROS and other free radicals under normal circumstances. In fact, under basal conditions, ROS are important signaling molecules. ROS in BECs help to regulate survival networks. Of note, ROS in BECs play a critical role in regulating vascular tone [12]. NO is generated by NOS, using L-arginine as a substrate. There are three different isoforms in mammals: eNOS, inducible NOS (iNOS), and neuronal NOS (nNOS). BECs only express the former two. NO regulates vascular tone by activating soluble guanylate cyclase in the vascular smooth muscle and helps to control mitochondrial oxygen consumption by inhibiting cytochrome c oxidase (complex IV). 

ROS can also regulate BEC angiogenic responses [6]. Angiogenesis involves new blood vessels sprouting from existing vasculature. This process is important during embryogenesis of course, but is also necessary in tissue repair. There is a fine balance between the beneficial and detrimental impact of ROS on angiogenesis. Small concentrations of hydrogen peroxide (H_2_O_2_) generated by Nox promoted BEC proliferation, migration, and tube formation [13,14]. ROS are able to stimulate induction of vascular endothelial growth factor (VEGF), a protein critical in angiogenesis [15]. Higher concentrations of H_2_O_2_ (>100 µM) have a detrimental effect by increasing BEC permeability and decreasing TJ protein localization [13]. 

Indeed, disruption of the BBB is a common response to oxidative stress [16]. TJs, as touched on above, can be disturbed due to oxidative stress. Changes in TJ protein levels and/or TJ protein cellular localization/trafficking are factors that contribute to BBB disruption. For example, the TJ protein occludin is pulled away from TJs (relocated away from its site of function at the cell membrane) during periods of increased oxidative stress [17]. Additionally, ROS can rearrange the BEC cytoskeleton, providing a molecular mechanism as to how ROS alter localization of TJ proteins, affecting BBB integrity [18]. The antioxidant ascorbate has been shown to reverse increases in endothelial cell barrier permeability by protecting against the microtubule destabilization [19] that results from high glucose-induced oxidative stress [20]. Matrix metalloproteinases (MMPs) are another common culprit in oxidative stress-induced BBB damage. MMPs are proteolytic enzymes that are capable of degrading extracellular matrix proteins and can cleave cell surface receptors. MMP-2 and MMP-9 are the main MMPs most closely linked to barrier permeability following oxidative insult [21]. MMPs, like MMP-9, are activated or upregulated in response to oxidative stress [22]. 

Within the NVU, astrocytes are the least susceptible to oxidative stress, which may help support the maintenance of the BBB during oxidative insult [23]. Astrocytes are also the source of glutathione, a key antioxidant. Pericytes are particularly sensitive to oxidative stress as, under disease conditions such as DM and AD, these cells are often the first cells of the NVU reported to die [10]. This increased susceptibility to oxidative stress is not well understood but could be due to a variety of reasons. First, pericytes are connected to BECs via gap junction connections, allowing direct cell–cell cytoplasm interactions and transfer of molecules, such as glucose. Second, it is possible that pericytes do not have the antioxidant capability to combat increases in oxidative stress compared to BECs or astrocytes. Treatment of pericytes with ascorbate prevents high glucose-induced apoptosis of this NVU cell type [24], suggesting added protection from enhanced antioxidant capacity. Lastly, the ratio of pericytes to BECs compared to other NVU cell types such as astrocytes and neurons is much smaller [25]. Therefore, a small loss of pericyte number could be much more detrimental to the NVU than a small loss of astrocytes. 

Autophagy, the ability for a cell to clear potentially toxic byproducts to generate nutrients needed to support metabolic reactions, is critical for endothelial cell homeostasis [26]. ROS are early inducers of autophagy during nutrient deprivation [27]. This protects cells from apoptosis during periods of low nutritional states. Alternatively, impairments in autophagy lead to oxidative stress in endothelial cells. Antioxidants can enhance autophagy in endothelial cells [28]. Intact autophagy can preserve endothelial function and promote eNOS activation [29]. 

When studying the antioxidant capacity or response of BECs in vitro, it is important to keep in mind that these cells can respond differently outside of the native environment. A recent study showed that various commercially available BEC lines had different responses to oxidative insult [30]. Additionally, co-culturing BECs with either astrocytes or pericytes can elicit a different antioxidant response as well. Astrocytes enhance the antioxidant activity of BECs through soluble, secreted factors [31]. The impact of oxidative stress on the BBB, as well as the contribution of the BBB to oxidative stress, can indeed be complicated. Deciphering cause and effect between these two systems is often hard to separate because BBB disruption can lead to oxidative stress, and oxidative stress can induce BBB disruption. 

## 3. Insulin, Oxidative Stress, and the BBB

CNS insulin is critical for its pleotropic role in metabolism as well as its ancestral role in acting as a growth factor. Acting as a metabolic factor, insulin is able to regulate feeding behavior as well as peripheral metabolism and glucose levels. CNS insulin can also regulate cognition and neuronal development [32]. Insulin crosses the BBB in an energy-dependent saturable manner [33]. Not only can insulin impact BECs themselves through intracellular signaling, but impairments in BEC insulin receptor signaling and/or transport of insulin across the BBB can lead to CNS insulin dysfunction [25,33]. 

**Figure 2:**
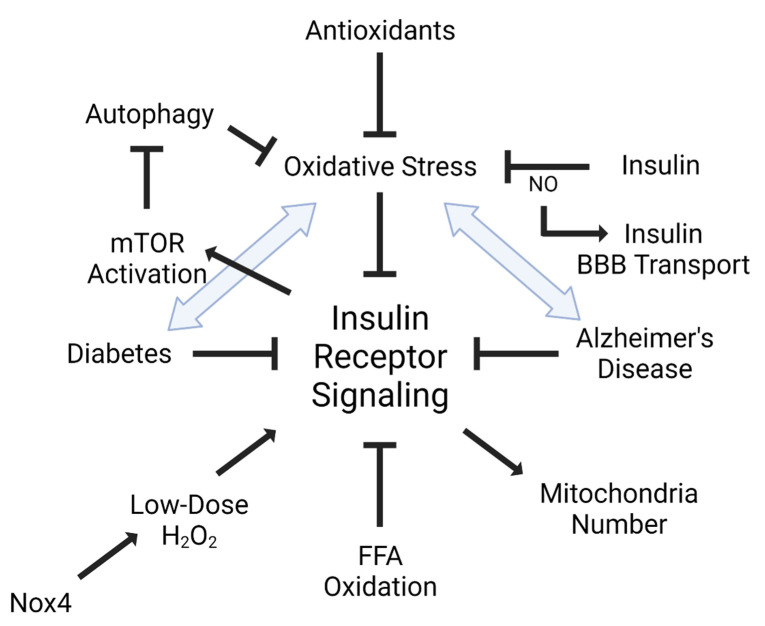
Interaction between insulin and oxidative stress. Insulin receptor signaling is tightly linked with oxidative stress. Dysregulated or impaired insulin receptor signaling is also defined as insulin resistance. Not only does oxidative stress, including free fatty acid (FFA) oxidation, impair insulin receptor signaling, but insulin receptor signaling can also activate mTOR to inhibit autophagy and oxidative stress. It is also known that insulin receptor signaling can regulate mitochondria number, which can be blocked by oxidative stress. Additionally, low doses of oxidative stress can have a positive impact on insulin receptor signaling, mediated by the NADPH oxidase, Nox4. Nitric oxide (NO) can regulate insulin BBB transport. Diseases implicated in insulin receptor signaling, such as diabetes mellitus (DM) and Alzheimer’s disease (AD), are heavily linked to oxidative stress. Figure generated using BioRender.

Insulin plays a role in oxidative stress, not only through its signaling networks, but also in insulin resistance (Figure 2). It has been shown that oxidative stress can lead to insulin resistance, particularly in the endothelium [34,35]. Additionally, increased oxidative stress markers are significantly associated with reduced insulin receptor activation [36]. Increased oxidation of free fatty acids in endothelial cells increases the production of superoxide by the mitochondrial electron transport chain leading to maladaptive insulin signaling [34]. ROS and oxidative stress are able to activate multiple serine kinase cascades [37], interrupting the insulin receptor signaling cascade. Alternatively, increased insulin levels can increase reactive oxygen species production and oxidative stress, accelerating insulin resistance [38]. Antioxidants such as alpha-lipoic acid, vitamin E, ascorbate, and glutathione are able to improve insulin sensitivity [39]. Insulin can act in an antioxidant manner by reversing high glucose-associated increases in ROS generation in peripheral endothelial cells [40].

As mentioned above, BECs interact with other cells of the NVU to maintain BBB structure and function, especially when it comes to oxidative stress. The role of the insulin receptor at the NVU has recently been investigated. Loss of the insulin receptor on astrocytes leads to decreases in mitochondrial number in neurons when the body is exposed to high glucose [41]. Alterations in mitochondrial number, as shown by Oldendorf et al. [7], can impact the degree of oxidative stress. This finding highlights two important characteristics about the BBB. First, as is well known, it links the periphery to the CNS through the impact of glucose, requiring high peripheral glucose to elicit a greater effect in the brain due to loss of the astrocytic insulin receptor. Second, it connects insulin receptor signaling on one cell type (astrocytes) to the communication with another cell type (neurons) and the potential impact on oxidative stress.

Another type of communication between cells of the NVU is neurovascular coupling. Neurovascular coupling is the mechanism by which cerebral blood flow is impacted through chemical and mechanical effects. Vascular tone is controlled by the NO pathway. NO bioavailability is largely affected by hyperglycemia-induced oxidative stress. Insulin can affect this pathway by stimulating enzymes needed to produce NO. Insulin BBB transport is increased under inflammatory conditions and further increased when NO synthesis is inhibited [42]. Under the same inflammatory conditions, NO released by endothelial NOS and inducible NOS indirectly stimulates insulin transport, whereas NO released by nNOS acts directly on BECs to block insulin transport in a region-specific manner [43]. These data support a direct link between insulin and NO, with a downstream impact on BBB transport.

Oxidative stress is also able to trigger insulin receptor signaling. Small oxidant molecules and reagents that generate H_2_O_2_ can mimic insulin action in adipocytes [44]. Indeed, insulin stimulation elicits a burst of H_2_O_2_ in target cells, enhancing insulin receptor phosphorylation. Recent molecular work found that the NADPH oxidase, Nox4, is responsible for the small burst of H_2_O_2_ production and is also able to stimulate insulin receptor phosphorylation and downstream signaling [45]. Nox4 is widely expressed in mouse BECs [46]. Whether similar signaling events occur in BECs remains to be determined.

Insulin can affect dietary lipid transport across the BBB, which can be a source for oxidative stress, as mentioned in the first section. Fatty acid transport protein 1 (FATP1) is the primary transporter for DHA across the BBB, accounting for nearly three-quarters of the total DHA uptake by BECs [47]. Insulin further increases DHA supply to the brain by promoting translocation of FATP1 to the cell surface. DHA has neuroprotective effects on cognitive function and memory in AD. While DHA is a target for lipid peroxidation, it is unknown whether dietary supplementation of DHA results in increased lipid peroxidation activity in brain. 

The impact on oxidative stress due to insulin at the BBB requires more direct investigation. Many of the studies listed above are indirectly related, suggesting ways in which oxidative stress could be implicated. Additionally, separating the impact of insulin compared to other factors involved in insulin resistance (i.e., high glucose) is difficult to parse out. Regardless, insulin and oxidative stress are clearly linked through insulin resistance, mitochondrial changes, NO signaling, and fatty acid transport. Apolipoprotein E (apoE) is one protein linked to insulin resistance, oxidative stress, and AD.

## 4. Apolipoprotein E Impact on BBB Oxidative Stress

ApoE exists in three isoforms in humans: apoE2, apoE3, apoE4. These three isoforms exist in known conformational and structural differences that lead to differential interactions with proteins and peptides, including amyloid β protein (Aβ) and hyperphosphorylated tau [48,49]. Individuals expressing apoE4 have increased levels of CNS oxidative stress [50]. Additionally, apoE4 carriers have increased Aβ in the vessel walls [51]. BEC exposure to Aβ dose-dependently increases oxidative stress [52]. The apoE4 isoform increases the activation of the cyclophilin A-MMP-9 pathway, leading to BBB disruption [53,54], which could be mediated by oxidative stress [55]. ApoE regulates production of NO [56]. Mice expressing apoE4 generate more NO compared to mice expressing apoE3 [57]. Additionally, apoE can act as an antioxidant at physiological levels, potentially through free metal sequestration. Mice lacking apoE are more susceptible to oxidation, suggesting a protective role against oxidative stress for apoE [58]. ApoE4 has the least functional antioxidant capacity, rendering this isoform as a poor protector from oxidative stress [59]. Development of oxidative stress, particularly in AD, could be due to expression of different apoE isoforms [60].

The various apoE isoforms have differential binding affinities to many proteins and peptides, such as Aβ and microtubules. Additionally, each apoE isoform has a different binding affinity to the insulin receptor [61,62]. ApoE3 binds stronger to the insulin receptor than apoE4 [63]. Aged mice expressing apoE4 have reductions in insulin receptor signaling and also respond less well to insulin stimulation compared to apoE3 mice [64]. In primary neurons, apoE4 interacts with the insulin receptor in a way that impairs trafficking, leading to impaired insulin signaling and insulin-stimulated mitochondrial respiration [64]. Since it is known that insulin receptor signaling is involved in oxidative stress, any disturbance in this signaling due to expression of the apoE4 isoform could contribute to changes in cellular oxidative stress. 

## 5. Diseases Associated with Insulin and Oxidative Stress

As reviewed above, oxidative stress occurs at the BBB by a number of mechanisms and pathways. Oxidative stress can result in BBB disruption and other dysfunctions of the BBB such as altered immune cell trafficking, toll-like receptor expression, and transporter functions. Oxidative stress at the BBB is often accompanied by inflammation and/or neuroinflammation, with oxidative stress being both a cause and result of inflammation [65,66,67]. A diversity of disease states, conditions, and therapeutics can cause oxidative stress at the BBB. These include induction by viral proteins [68], methamphetamine [66], highly active anti-retroviral therapy [69], obesity [70], and aging [10]. Here, we will focus on two conditions: DM and AD.

### 5.1. Diabetes Mellitus (DM)

DM is defined by the body’s inability to properly respond to insulin, resulting in hyperglycemia and abnormal metabolism of carbohydrates. Disruption of the BBB occurs in diabetic humans [71], rhesus monkeys [72], and in rodent models of both insulinopenic and insulin-resistant DM [73,74,75]. Many other alterations in BBB function occur with diabetes, including changes in the brain-to-blood (efflux) transporters P-glycoprotein (P-gp) and LDL receptor-related protein 1 (LRP-1). Changes in the receptor for advanced glycation end products (RAGE), MMPs, the choline transporter, NMDA-dependent vasodilation, immune cell trafficking, and ascorbate transport all occur at the BBB in DM or models of DM [76]. 

Several mechanisms have been shown to be involved in the oxidative stress of the BBB in DM. These include pericyte loss from excessive glycolysis, production of methylglyoxal, and involvement of nuclear factor erythroid 2-related factor 2 (Nrf2), a transcription factor involved in the production of antioxidant proteins.

The major cause of blindness in the West is diabetic retinopathy, which is a disruption of the blood–retina barrier (BRB). As the retina is a cranial nerve, and thus part of the CNS, the BRB is often viewed as a specialized arm of the BBB [77]. The mechanism of diabetic retinopathy has long been understood to be a hyperglycemic-induced loss of pericytes [78,79], cells that are key to BBB induction and maintenance [80]. Work with the BBB has shown that oxidative stress induced by excess glycolysis in pericytes underlies the demise of first pericytes and then BBB integrity. Blockade of glycolysis with mitochondrial carbonic anhydrase inhibitors reduces oxidative stress, preserves pericytes, and protects against BBB disruption [81,82].

Methylglyoxal, a derivative of pyruvic acid, is a highly reactive and cytotoxic compound that can interact with other molecules to form advanced glycation end products (AGEs). AGEs, in turn, result in oxidative stress and loss of BBB integrity [83]. Production of methylglyoxal is increased in hyperglycemia and has been associated with diabetic atherosclerosis and worsening of neuropathic pain. Methylglyoxal can react with BEC TJ proteins, resulting in BBB disruption [84,85]. Methylglyoxal is typically detoxified by glutathione, and treatment with N-acetylcysteine restores glutathione levels and protects against methylglyoxal-induced BBB disruption. 

Nrf2 activation can improve insulin sensitivity in a mouse model of diabetes [86]. Nrf2 gene expression in the CNS is most predominant in BECs and microglia [46]. Studies have shown a protective role for Nrf2 in preserving the BBB from diabetes-related oxidative stress. BBB disruption inversely correlated in one study with Nrf2 expression, with lower expression of Nrf2 being associated with higher levels of BBB disruption [87]. 

Treating oxidative stress at the BBB often begins with addressing hyperglycemia. Other substances that have been shown to reduce oxidative stress and to preserve BBB function in DM or models of DM include epoxyeicosatrienoic acids [88]. In a type II DM model, telmisartan reduces oxidative stress, preserves BBB integrity, and maintains TJ protein expression [89].

### 5.2. Alzheimer’s Disease (AD)

AD is pathologically defined by the presence of Aβ plaques and hyperphosphorylated tau tangles. Oxidative stress in the AD brain, including its microvasculature, is high [90]. One cause of oxidative stress is Aβ. Knocking down Aβ levels by treating with an antisense directed against amyloid precursor peptide results in a reduction in oxidative stress in the brain [91]. The methionine at position 35 is a free radical source, producing oxidative stress. In support of this, a mutated form of Aβ with a non-reducing amino acid substituted for methionine at the 35 position does not produce neurotoxicity or oxidative stress [92]. Aβ induces oxidative stress at the BBB, resulting in decreased TJ protein expression [52]. In the aged SAMP8, which is used as a mouse model of AD, treatment with the potent antioxidant alpha lipoic acid and N-acetylcysteine reverses the oxidative stress and memory impairment seen in that model [93].

Recent evidence has shown that the BBB is mildly disrupted in AD and that this disruption is associated with pericyte dysfunction [94]. Pericytes are key to the induction and maintenance of the BBB and their loss is associated with BBB dysfunction in AD and DM as well as several other diseases [95]. Pericytes are very sensitive to oxidative stress and their loss has been associated with oxidative stress in at least some conditions. Although Aβ can affect both TJ protein expression [96] and pericyte viability [97], evidence is that pericyte loss occurs very early in the course of the disease, before the onset of mild cognitive impairment or positive Pittsburgh compound B (PIB) scans, which detect Aβ [94]. Therefore, pericyte loss and BBB disruption occur very early in the course of AD. 

Just as in DM, studies have implicated the loss of Nrf2, with increased susceptibility to BBB dysfunction related to obesity, aging, and AD [98]. Glycogen synthase kinase-3 (GSK) antisense in SAMP8 protects against oxidative stress via an Nrf2 pathway [99]. Other work emphasizes a major role for microglial inflammation resulting in oxidative stress at the BBB [100].

As discussed above, two major efflux (brain-to-blood) transporters located at the BBB are P-gp and LRP-1. These transporters have many ligands, including Aβ. Clearance of Aβ from the brain is predominantly dependent on these pumps, both of which are impaired in AD and the AD models SAMP8 and Tg7645 [101,102,103]. The neurovascular hypothesis of Zlokovic states that the impaired efflux of Aβ from the brain contributes to Aβ accumulation and the progression of AD [103]. The likely role of oxidative stress as underlying the impairment of P-gp has recently been reviewed [104]. In the hippocampus of subjects with AD, the level of LRP-1 is not altered, but the amount LRP-1 that is oxidized is greatly increased [105]. Treatment with N-acetylcysteine reverses the inflammation-induced impairment of Aβ efflux [106]. Many xenobiotics are also substrates of P-gp, and so decreased P-gp activity may place AD patients at increased risk of drug-related neurotoxicity. 

Inhibition of the mammalian target of rapamycin (mTOR) protects the vasculature from aging by reducing oxidative stress [107]. Rapamycin, an mTOR inhibitor, can preserve the BBB in a mouse model of AD by limiting BBB disruption, upregulating TJ proteins, and downregulating MMPs [108]. Recently, there has been a push to test the clinical efficacy of rapamycin in AD [109]. We recently found that, while rapamycin did not impact insulin transport across the BBB, it did affect insulin binding at the BBB, which could impact downstream insulin receptor signaling [110].

## 6. Conclusions

Oxidative stress can occur in any organ throughout the body and each organ has its own unique response to combat the production of oxidative stress. For example, oxidative stress is known to impact the gut microbiome, which can indirectly lead to alterations at the BBB through release of circulatory factors and have been linked to neurodegenerative and metabolic disease [111,112]. We chose to focus this review on the BBB and accompanying BECs, due to the interactions between insulin resistance and two primary diseases in which insulin resistance is implicated: DM and AD. We recognize one limitation of our review, which is the limited discussion regarding transport of antioxidants across the BBB. This topic deserves an independent review as there are a number of endogenous and exogenous antioxidants or antioxidant-like compounds, such as polyphenols, that require BBB transport to enter the CNS, utilizing different transport systems and stereoselectivity. Phenolic compound and other antioxidant BBB transport has been reviewed elsewhere [113,114]. Additionally, as antioxidant defense systems vary greatly between humans and basic laboratory models, covering BBB transport systems and distinguishing between different models are beyond the scope of this review. Instead, we focused our review on the BECs themselves, rather than transport across this cell type. We presented the literature regarding BECs antioxidant defense systems and the generation of oxidative stress within BECs. We discussed ways in which insulin can mediate or contribute to oxidative stress at the BBB and the involvement of apoE. 

Lastly, we highlighted two diseases affected by oxidative stress and involved in insulin resistance. Although AD and DM are very different diseases, one affecting cognition and the other peripheral glucose metabolism, they share many mechanistic similarities. Diabetes, hyperglycemia, and obesity are risk factors for AD and other cognitive deficits, both diseases have disruptions and other dysfunctions at the BBB, and both can be associated with insulin resistance, one mainly in the peripheral tissues and the other mainly at brain tissues. These similarities in symptoms may be mediated by the similarities in their underlying disease mechanisms. For both diseases, one of the most prominent characteristics is that of oxidative stress involving Nrf2, neuroinflammation, pericyte loss, and similar alterations in BBB functions [115]. These connections suggest that, not only may both diseases be treatable by similar drugs [116], but they may explain why metabolic drugs commonly used to treat diabetes hold potential for the treatment of AD as well. Regardless of how oxidative stress may be involved in these diseases, targeting oxidative stress or insulin resistance can clearly improve the structure and function of the BBB.

## Abbreviations

Aβ	amyloid beta
AD	Alzheimer’s disease
AGE	advanced glycation end product
ApoE	apolipoprotein E
BBB	blood–brain barrier
BEC	brain endothelial cell
BM	basement membrane
BRB	blood–retinal barrier
CNS	central nervous system
DHA	docosahexaenoic acid
DM	diabetes mellitus
FATP	fatty acid transport protein
FFA	free fatty acid
LRP-1	low density lipoprotein (LDL) receptor-related protein 1
MMP	matrix metalloproteinase
mTOR	mammalian target of rapamycin
NADPH	nicotinamide adenine dinucleotide phosphate
NO	nitric oxide
NOS	nitric oxide synthase (i- inducible, e- endothelial, n- neuronal)
Nox	NADPH oxidase
Nrf2	nuclear factor erythroid 2-related factor 2
NVU	neurovascular unit
Pgp	P-glycoprotein
RAGE	receptor for advanced glycation end product
RNS	reactive nitrogen species
ROS	reactive oxygen species
TJ	tight junction
VEGF	vascular endothelial growth factor
